# Supplementary material for: ProSiteHunter: A Unified Framework for Sequence‐Based Prediction of Protein‐Nucleic Acid and Protein‐Protein Binding Sites
Source: Adv Sci (Weinh). 2026 Jun 2:e75931. Online ahead of print. doi: 10.1002/advs.75931 (PMC13336437; doi:10.1002/advs.75931)
Supplement: Supplementary file 1 — Supporting File: advs75931‐sup‐0001‐SuppMat.docx. [file ADVS-9999-e75931-s001.docx]

**Supplementary Information for**

**ProSiteHunter: A unified framework for sequence-based prediction of protein-nucleic acid and protein-protein binding sites**

Dongliang Hou^1^, Qihang Zhen^1^, Zexin Lv^1^, Xinyue Cui^1^, Suhui Wang^1^, Minghua Hou^1^, Zhan Zhou^2,∗^, Xiaogen Zhou^1,∗^, Guijun Zhang^1,∗^

1 College of Information Engineering, Zhejiang University of Technology, 288 Liuhe Road, 310023 HangZhou, China.

2 State Key Laboratory of Advanced Drug Delivery and Release Systems and Innovation Institute for AI in Medicine, College of Pharmaceutical Sciences, Zhejiang University, Hangzhou, China

∗ Correspondence should be addressed to Guijun Zhang (Email: [zgj@zjut.edu.cn](mailto:zxg@zjut.edu.cn)), Xiaogen Zhou (Email: [zxg@zjut.edu.cn](mailto:zxg@zjut.edu.cn)) and Zhan Zhou (Email: [zhanzhou@zju.edu.cn](mailto:zxg@zjut.edu.cn))

**Table S1.** Detailed description of input features for the network.

| **Feature** | | **Description** | **Dimensions** |
| --- | --- | --- | --- |
| **Evolutionary features** | **SiteT5** | SiteT5 is an enhanced protein language model that retains the first 50 MSA data per sequence for fine-tuning of ProtT5-XL-UniRef50 | L×1024 |
|  | **ProstT5** | ProstT5 selected 17M high-quality protein sequence-structure pairs and fine-tuned ProtT5-XL-UniRef50 via a 3Di coding format | L×1024 |
| **Statistical**  **features** | **BLOSUM62 Matrix** | Blosum62 is based on evolutionarily conserved statistical properties and is used to measure the probability of substitution between different amino acids in protein sequence alignment. | L×24 |
|  | **Amino acid propensity** | Through systematic statistical analysis of the regions labeled as binding sites in the training set, we found significant differences in amino acid composition between different types of protein binding sites. | L×1 |
|  | **Physicochemical properties** | Five physicochemical properties relevant to site prediction tasks. steric hindrance parameters, polarizability, amino acid side chain volume, hydrophobicity, and isoelectric point | L×5 |
| **Geometric features** | **secondary structure** | The flexible loop regions are more likely to interact with other macromolecules. Therefore, eight more detailed secondary structures have been selected for description. | L×8 |
|  | **Positional**  **encoding** | A symmetrical normalized position code that measures the degree of symmetry of amino acids in a sequence. | L×1 |
|  | **Relative solvent accessibility** | Relative solvent accessibility can clearly distinguish between surface and buried residues, and the addition of this feature narrows the range of binding sites for prediction tasks. | L×1 |

**Table S2.** The number of positive and negative samples in the protein-DNA binding site dataset and the protein-RNA binding site dataset

| **Datasets** | **protein-DNA**  **(train)** | **protein-DNA**  **(test)** | **protein-RNA**  **(train)** | **protein-RNA**  **(test)** |
| --- | --- | --- | --- | --- |
| binding sites | 14479 | 2240 | 14609 | 2031 |
| non-binding sites | 145404 | 35275 | 122290 | 35314 |

**Table S3.** Compare the performance of ProSiteHunter, CLAPE-DB, iDRNA-ITF, and DRNApred on the protein-DNA binding site test set (Test129).

| **Method** | **ROCAUC** | **PRAUC** | **F1** | **MCC** |
| --- | --- | --- | --- | --- |
| ProSiteHunter | 0.917 | 0.497 | 0.511 | 0.486 |
| CLAPE-DB | 0.865 | 0.394 | 0.380 | 0.367 |
| iDRNA-ITF | 0.834 | 0.400 | 0.421 | 0.386 |
| DRNApred | 0.692 | 0.143 | 0.214 | 0.150 |

**Table S4** Compare the performance of ProSiteHunter, CLAPE-RB, iDRNA-ITF, and DRNApred on the protein–RNA binding site test set (Test117).

| **Method** | **ROCAUC** | **PRAUC** | **F1** | **MCC** |
| --- | --- | --- | --- | --- |
| ProSiteHunter | 0.852 | 0.320 | 0.375 | 0.340 |
| CLAPE-RB | 0.788 | 0.179 | 0.281 | 0.240 |
| iDRNA-ITF | 0.702 | 0.186 | 0.276 | 0.231 |
| DRNApred | 0.662 | 0.124 | 0.175 | 0.122 |

**Table S5** **Performance comparison of ProSiteHunter and other methods on the public benchmark dataset Test355 for protein binding site prediction.**

| Methods | ROCAUC | PRAUC | F1 | MCC |
| --- | --- | --- | --- | --- |
| ProSiteHunter | 0.883 | 0.581 | 0.536 | 0.478 |
| Seq-InSite | 0.860 | 0.527 | 0.523 | 0.458 |
| D-PPIsite* | 0.823 | 0.448 | 0.460 | 0.387 |
| PITHIA | 0.763 | 0.330 | 0.381 | 0.297 |
| ISPRED | 0.718 | 0.324 | 0.327 | 0.226 |
| LORIS* | 0.637 | 0.203 | 0.241 | 0.137 |
| PSIVER* | 0.583 | 0.155 | 0.177 | 0.065 |

* Indicating that the results are directly cited from the literature

**Table S6** **Performance comparison of ProSiteHunter and other methods on the public benchmark dataset Test70 for protein binding site prediction.**

| Methods | ROCAUC | PRAUC | F1 | MCC |
| --- | --- | --- | --- | --- |
| ProSiteHunter | 0.783 | 0.483 | 0.499 | 0.355 |
| Seq-InSite | 0.762 | 0.436 | 0.447 | 0.311 |
| EnsemPPIS | 0.719 | 0.405 | 0.440 | 0.277 |
| ISPRED | 0.712 | 0.351 | 0.431 | 0.261 |
| PITHIA | 0.689 | 0.367 | 0.406 | 0.218 |
| DELPHI | 0.690 | 0.356 | 0.418 | 0.236 |
| DeepPPISP | 0.667 | 0.322 | 0.397 | 0.206 |

**Table S7** Compare the performance of ProSiteHunter, CALIBER, Bepipred-3.0, and SEMA2.0-1D on the antibody-antigen binding site test set (Test101).

| **Method** | **ROCAUC** | **PRAUC** | **F1** | **MCC** |
| --- | --- | --- | --- | --- |
| ProSiteHunter | 0.787 | 0.282 | 0.310 | 0.250 |
| CALIBER | 0.756 | 0.245 | 0.277 | 0.216 |
| Bepipred-3.0 | 0.733 | 0.211 | 0.272 | 0.214 |
| SEMA2.0-1D | 0.730 | 0.187 | 0.223 | 0.179 |

**
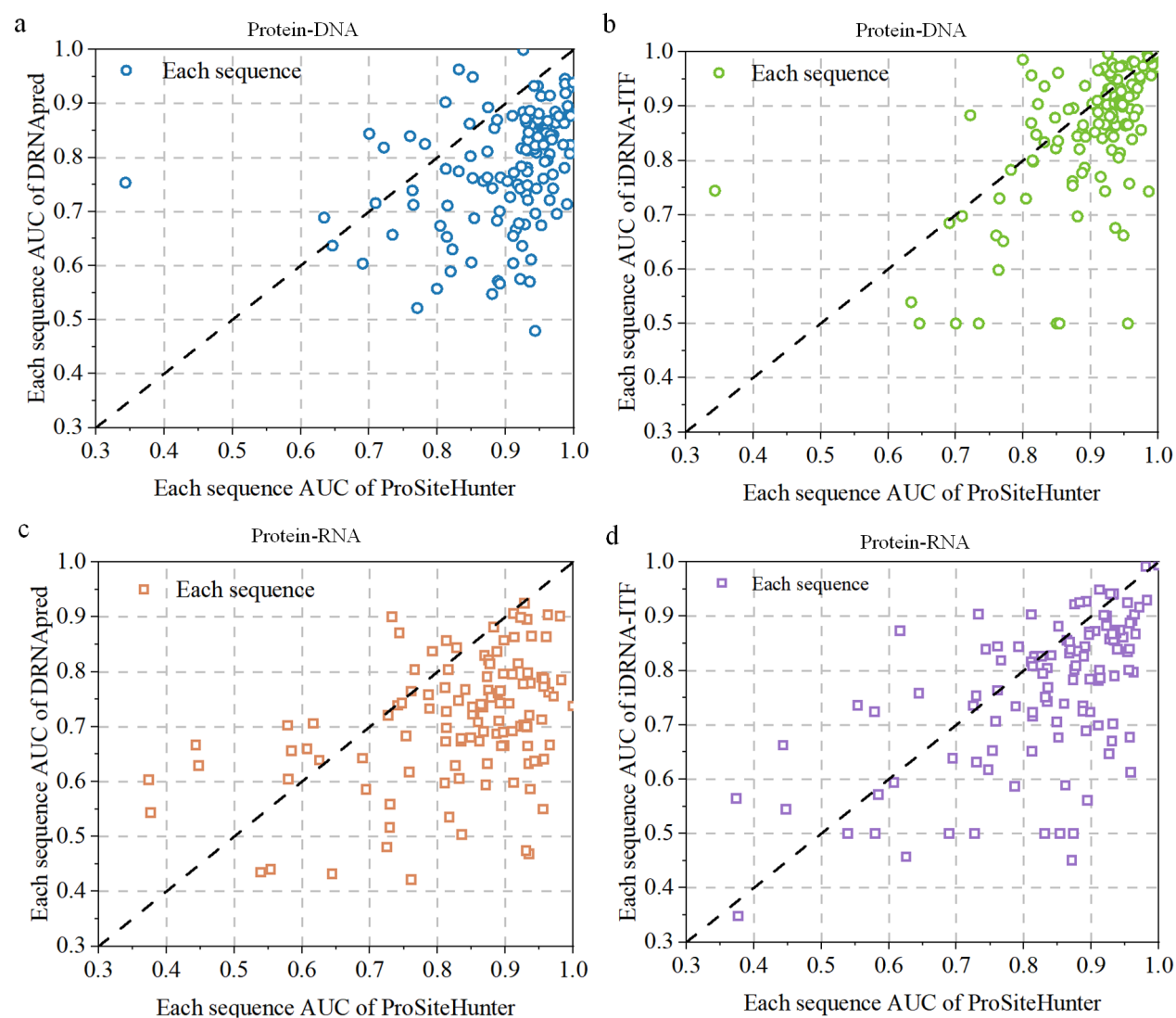
**

**Fig. S1 a,** Scatter plots display the sequence-wise AUC values of ProSiteHunter versus DRNApred for protein-DNA binding. **b,** Scatter plots display the sequence-wise AUC values of ProSiteHunter versus iDRNA-ITF for protein-DNA binding. **c,** Scatter plots display the sequence-wise AUC values of ProSiteHunter versus DRNApred for protein-RNA binding. **d,** Scatter plots display the sequence-wise AUC values of ProSiteHunter versus iDRNA-ITF for protein-RNA binding.


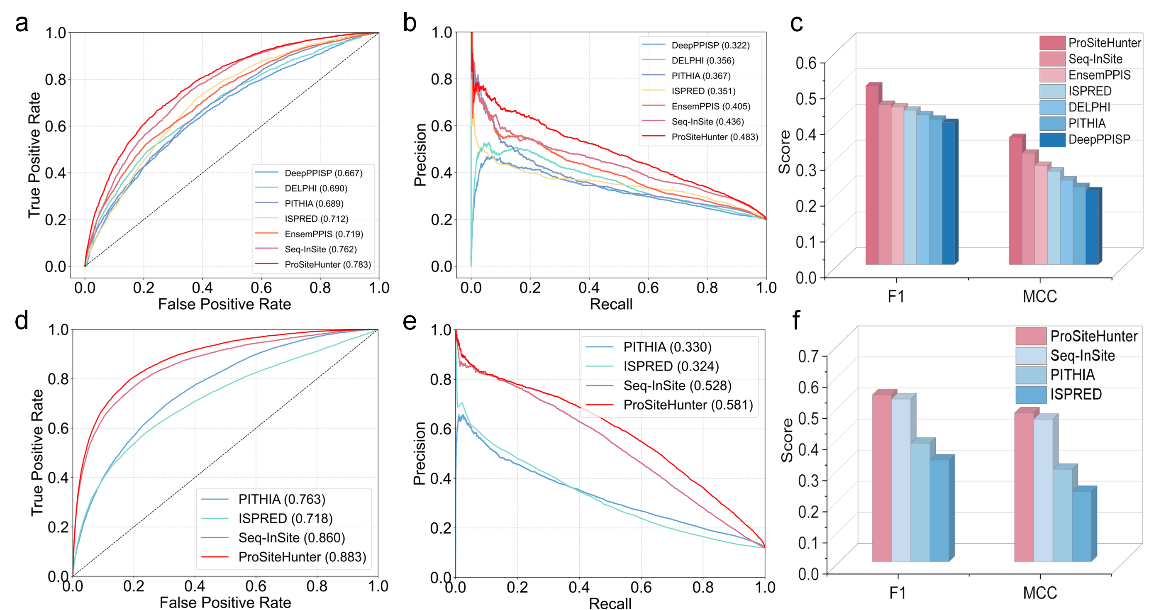


**Fig. S2 a,** ROC AUC curves of ProSiteHunter on the Test70 benchmark dataset for protein binding site prediction. **b,** PR AUC curves of ProSiteHunter on the Test70 benchmark dataset for protein binding site prediction. **c,** Performance comparison of ProSiteHunter and baseline methods in terms of F1 score and MCC on the Test70 benchmark dataset.

As shown in **Fig. S3** We conducted a comparative experiment by removing the RSA feature (w/o RSA) and comparing the performance with ProSiteHunter (which uses RSA).

Taking the protein-DNA binding site prediction task as an example, although removing the RSA feature leads to a slight decrease in performance, the overall impact is not significant. Specifically, ROCAUC, PRAUC, F1-score, and MCC decrease by 0.35%, 1.16%, 1.57%, and 2.47%, respectively. Similar trends are observed across the other tasks. These results indicate that, while RSA contributes to the performance of ProSiteHunter, it is not the sole factor driving the observed improvements. Even in the absence of the RSA feature, the model is still able to maintain competitive performance.


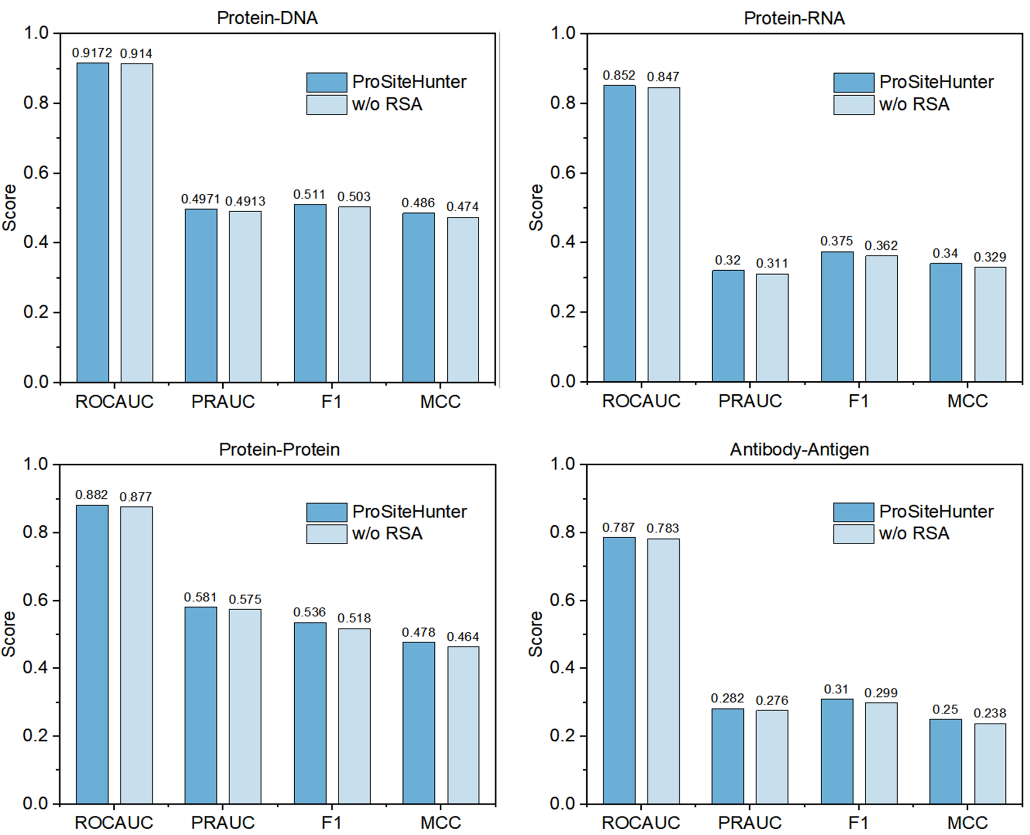


**Fig. S3 Performance comparison of ProSiteHunter with and without the RSA feature across four binding site prediction tasks**


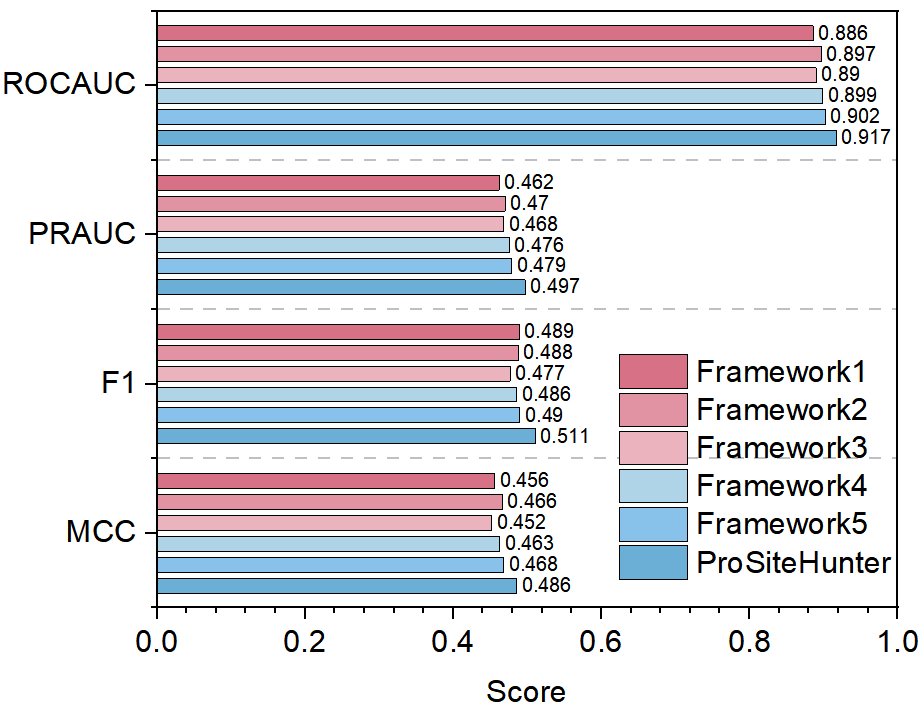


**Fig. S4 Performance Comparison of Models with Different Q/K/V Allocation Strategies.**

**Table S8. Explanation of Q/K/V Assignment Schemes Across Different Frameworks.**

| **Framework1** | **Context-Aware Encoder as Q**  **Scale-Aware Encoder as K**  **Importance-Aware Encoder as V** |
| --- | --- |
| **Framework2** | **Importance-Aware Encoder as Q**  **Scale-Aware Encoder as K**  **Context-Aware Encoder as V** |
| **Framework3** | **Scale-Aware Encoder as Q**  **Importance-Aware Encoder as K**  **Context-Aware Encoder as V** |
| **Framework4** | **Importance-Aware Encoder as Q**  **Context-Aware Encoder as K**  **Scale-Aware Encoder as V** |
| **Framework5** | **Context-Aware Encoder as Q**  **Importance-Aware Encoder as K**  **Scale-Aware Encoder as V** |
| **ProSiteHunter** | **Scale-Aware Encoder as Q**  **Context-Aware Encoder as K**  **Importance-Aware Encoder as V** |

**Table S9 The number of sequences in each amino acid length range in the benchmark test sets of the four tasks.**

| length  Task type | Below 100 | 100-200 | 200-300 | 300-400 | 400-500 | Above 500 | Total sequences |
| --- | --- | --- | --- | --- | --- | --- | --- |
| Protein-DNA | 14 | 41 | 32 | 15 | 8 | 19 | 129 |
| Protein-RNA | 13 | 33 | 19 | 19 | 14 | 19 | 117 |
| Protein-Protein | 27 | 87 | 106 | 81 | 36 | 18 | 355 |
| Antibody-Antigen | 10 | 27 | 30 | 18 | 6 | 10 | 101 |


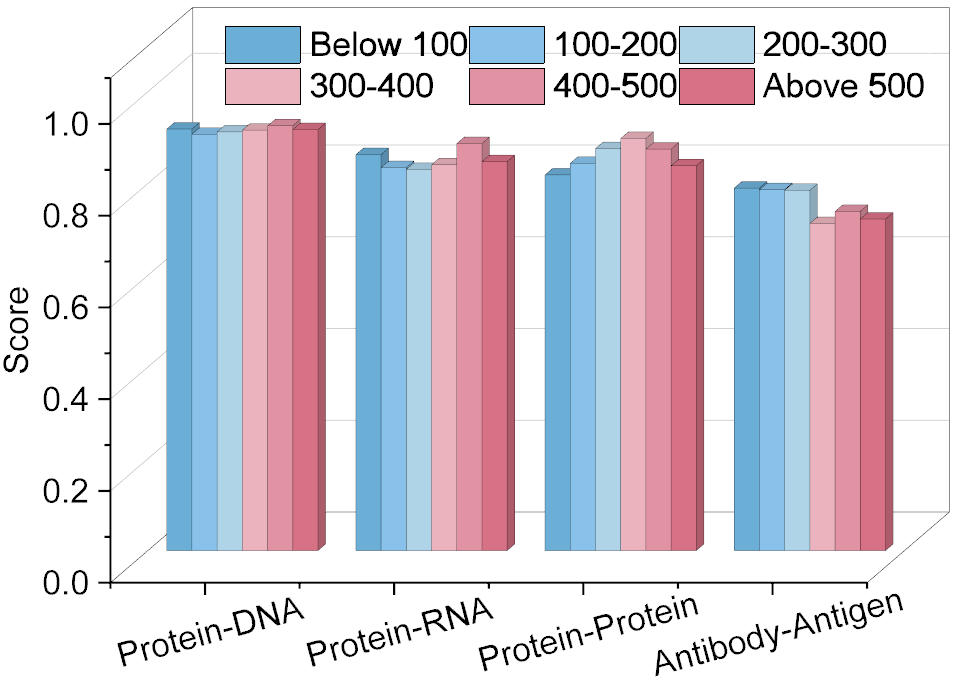


**Fig. S5 Comparison of ROC AUC performance of four tasks divided by sequence length.**

**Table S10 ROC AUC values for sequences across amino acid length ranges in the benchmark test set for the four tasks.**

| length  Task type | Below 100 | 100-200 | 200-300 | 300-400 | 400-500 | Above 500 | avg AUC |
| --- | --- | --- | --- | --- | --- | --- | --- |
| Protein-DNA | 0.919 | 0.908 | 0.913 | 0.917 | 0.926 | 0.918 | 0.917 |
| Protein-RNA | 0.863 | 0.835 | 0.831 | 0.841 | 0.888 | 0.848 | 0.851 |
| Protein-Protein | 0.819 | 0.844 | 0.876 | 0.900 | 0.875 | 0.839 | 0.859 |
| Antibody-Antigen | 0.790 | 0.787 | 0.786 | 0.713 | 0.740 | 0.723 | 0.757 |

**Table S11 Performance comparison under 10 different random seeds for the Protein-DNA binding site prediction task**

| seed | 10 | 20 | 30 | 40 | 50 | 60 | 70 | 80 | 90 | 100 |
| --- | --- | --- | --- | --- | --- | --- | --- | --- | --- | --- |
| ROCAUC | 0.915 | 0.917 | 0.914 | 0.914 | 0.919 | 0.915 | 0.914 | 0.914 | 0.917 | 0.916 |
| PRAUC | 0.493 | 0.493 | 0.476 | 0.491 | 0.496 | 0.496 | 0.497 | 0.494 | 0.495 | 0.491 |
| F1 | 0.506 | 0.499 | 0.496 | 0.500 | 0.509 | 0.510 | 0.506 | 0.500 | 0.507 | 0.506 |
| MCC | 0.481 | 0.470 | 0.472 | 0.474 | 0.485 | 0.483 | 0.480 | 0.477 | 0.480 | 0.479 |

**Table S12 Performance comparison under 10 different random seeds for the Protein-RNA binding site prediction task**

| seed | 10 | 20 | 30 | 40 | 50 | 60 | 70 | 80 | 90 | 100 |
| --- | --- | --- | --- | --- | --- | --- | --- | --- | --- | --- |
| ROCAUC | 0.849 | 0.831 | 0.847 | 0.840 | 0.843 | 0.853 | 0.843 | 0.836 | 0.848 | 0.837 |
| PRAUC | 0.315 | 0.292 | 0.310 | 0.294 | 0.308 | 0.320 | 0.305 | 0.277 | 0.308 | 0.287 |
| F1 | 0.365 | 0.322 | 0.373 | 0.360 | 0.371 | 0.376 | 0.362 | 0.340 | 0.368 | 0.357 |
| MCC | 0.331 | 0.307 | 0.336 | 0.321 | 0.333 | 0.341 | 0.324 | 0.300 | 0.332 | 0.320 |

**Table S13 Performance comparison under 10 different random seeds for the Protein-Protein binding site prediction task**

| seed | 10 | 20 | 30 | 40 | 50 | 60 | 70 | 80 | 90 | 100 |
| --- | --- | --- | --- | --- | --- | --- | --- | --- | --- | --- |
| ROCAUC | 0.880 | 0.880 | 0.881 | 0.882 | 0.880 | 0.881 | 0.881 | 0.881 | 0.882 | 0.881 |
| PRAUC | 0.578 | 0.578 | 0.583 | 0.578 | 0.577 | 0.579 | 0.576 | 0.579 | 0.581 | 0.580 |
| F1 | 0.530 | 0.524 | 0.532 | 0.529 | 0.532 | 0.530 | 0.521 | 0.527 | 0.523 | 0.528 |
| MCC | 0.472 | 0.467 | 0.473 | 0.472 | 0.473 | 0.472 | 0.464 | 0.469 | 0.467 | 0.470 |

**Table S14 Performance comparison under 10 different random seeds for the Antibody-Antigen binding site prediction task**

| seed | 10 | 20 | 30 | 40 | 50 | 60 | 70 | 80 | 90 | 100 |
| --- | --- | --- | --- | --- | --- | --- | --- | --- | --- | --- |
| ROCAUC | 0.774 | 0.783 | 0.778 | 0.766 | 0.789 | 0.772 | 0.779 | 0.773 | 0.770 | 0.774 |
| PRAUC | 0.271 | 0.266 | 0.275 | 0.250 | 0.298 | 0.281 | 0.301 | 0.273 | 0.284 | 0.268 |
| F1 | 0.308 | 0.309 | 0.305 | 0.258 | 0.323 | 0.325 | 0.331 | 0.311 | 0.320 | 0.308 |
| MCC | 0.247 | 0.246 | 0.246 | 0.218 | 0.264 | 0.263 | 0.271 | 0.247 | 0.258 | 0.248 |


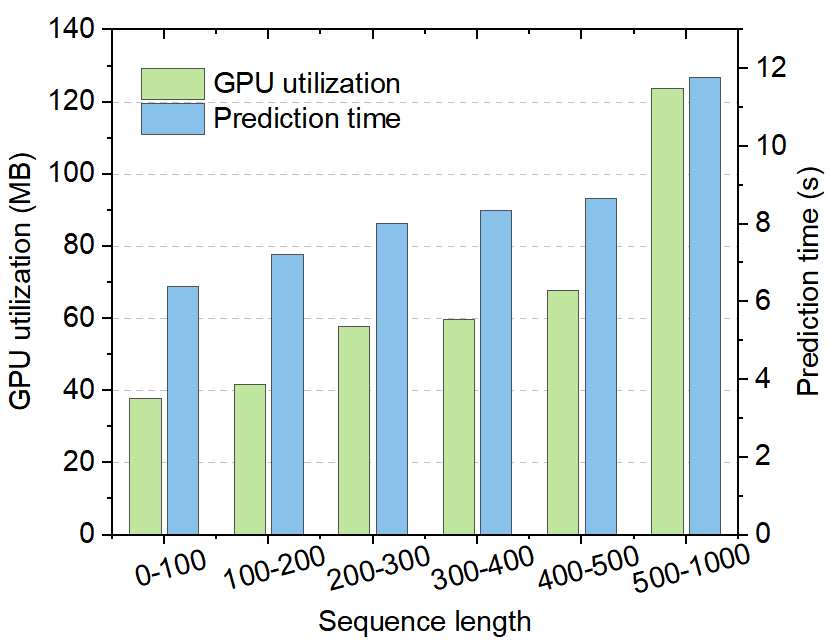


**Fig. S6 Computational cost analysis of the model across different sequence lengths**

**Table S15 Practical Computational Cost Analysis of the Model Across Different Sequence Lengths**

| Sequence length | GPU memory usage | Prediction time | Total parameters |
| --- | --- | --- | --- |
| 0-100 | 37.8MB | 6.39s | 1617834 |
| 100-200 | 41.8MB | 7.23s |  |
| 200-300 | 57.8MB | 8.01s |  |
| 300-400 | 59.8MB | 8.36s |  |
| 400-500 | 67.8MB | 8.67s |  |
| 500-1000 | 123.8MB | 11.78s |  |


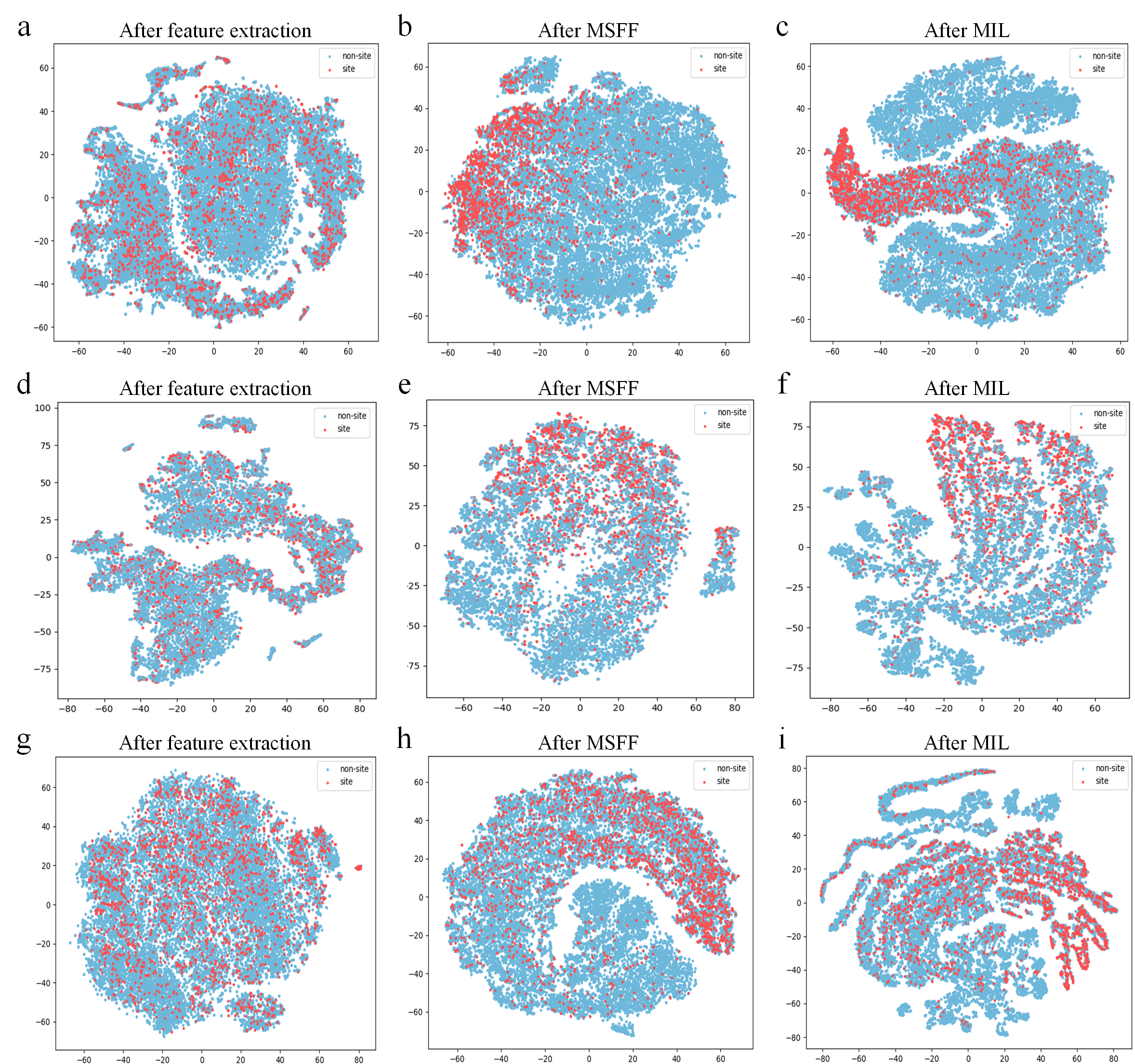


**Fig. S7 a-c,** Use the t-SNE graph to map the high-dimensional tensor after feature extraction, MSFF module, and MIL module into a two-dimensional space to describe the relationship between protein-RNA sites and non-sites. **d-f,** Use the t-SNE graph to map the high-dimensional tensor after feature extraction, MSFF module, and MIL module into a two-dimensional space to describe the relationship between protein-protein sites and non-sites. **g-i,** Use the t-SNE graph to map the high-dimensional tensor after feature extraction, MSFF module, and MIL module into a two-dimensional space to describe the relationship between antibody-antigen sites and non-sites.

**
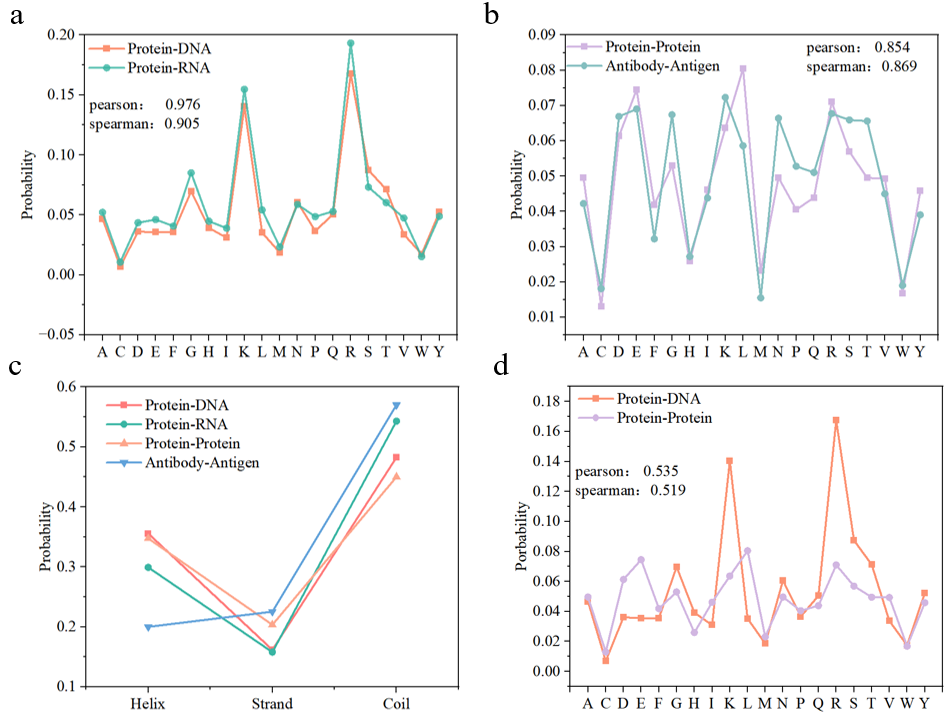
**

**Fig. S8 a,** Amino acid frequency statistics at protein-DNA binding sites and protein-RNA binding sites. **b,** Amino acid frequency statistics at protein-protein binding sites and antibody-antigen binding sites. **c,** the secondary structure composition of protein-DNA, protein-RNA, protein-protein, and antibody-antigen binding sites. **d,** Amino acid frequency statistics at protein-DNA binding sites and protein-protein binding sites.

We then analyzed the amino acid trends for different types of binding sites statistically based on the training set data. The binding sites of DNA-binding proteins and RNA-binding proteins are highly consistent in amino acid composition, with a Pearson correlation coefficient of 0.976 and a Spearman correlation coefficient of 0.905, which may indicate that there is a similarity in the binding patterns between the two types of proteins and nucleic acid molecules. Similarly, the amino acid composition at the protein-protein interaction sites and the antibody-antigen binding sites (epitopes) was highly similar (Pearson = 0.854, Spearman = 0.869), suggesting that despite the specificity of antibody antigens, their binding patterns are the same as those of conventional protein-protein interactions. Specific details will be provided in **Fig. S8a, S8b**

In the task of predicting four types of binding sites (statistically based on the training set data), we found that the prediction difficulty of antibody-antigen binding sites and RNA-binding protein sites was significantly higher than that of DNA-binding protein sites and protein-protein interaction sites. This phenomenon seems paradoxical, as the previous analysis has shown that these binding sites are highly similar in amino acid composition (RNA-binding protein sites and DNA-binding protein sites, protein-protein interaction sites, and antibody-antigen binding sites) and should theoretically exhibit similar binding patterns. To explore the potential reasons for this difference, we conducted a more in-depth analysis of the data. **Fig. S8c** statistically analyzed the secondary structure composition of protein-DNA, protein-RNA, protein-protein, and antibody-antigen binding sites, and the results showed that the loop was dominant among the four binding sites (accounting for 45%-57%), indicating that it plays a key role in the process of sites recognition. It is worth noting that RNA-binding regions and antibody-binding epitope regions exhibit the highest proportion of loop structures. (54.3% and 57.2%, respectively), and these two types of binding sites also show higher prediction difficulty, which may indicate that the high flexibility of loops may be an important factor leading to the decrease in prediction accuracy. In addition, unlike the other three types of binding sites tasks, antigen sites (epitopes) exhibit a unique secondary structure distribution characteristic: the proportion of $\beta$ fold is higher than that of $\alpha$ helix. We speculate that this phenomenon may be related to the fact that the hypervariable loop region of the epitope accounts for nearly 60% of the total, and it needs to provide a stable structural framework $\beta$ folding to maintain its conformational stability.

A comparative analysis of the amino acid tendencies of protein-DNA and protein-protein binding sites (**Fig. S8d)** shows that the two classes of binding sites exhibit significantly different binding preferences (Pearson:0.515, Spearman:0.519), suggesting that different types of protein-macromolecule interactions may follow different recognition mechanisms.


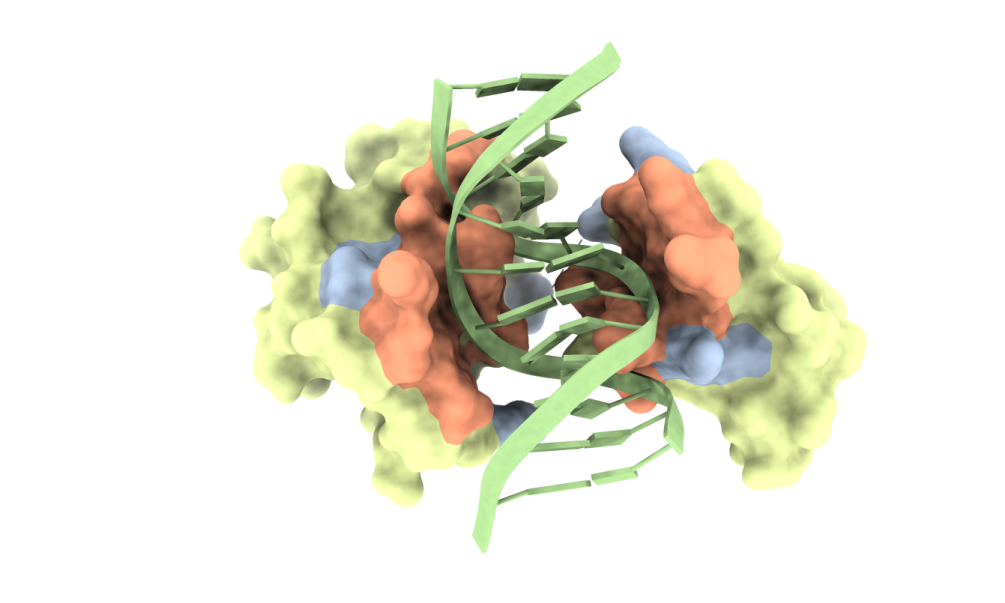


**Fig. S9** This protein (PDB ID: 5FD3) is a complex of DNA (green) and the Lin54 tesmin domain (yellow). The orange portion represents the actual binding site, while the blue and orange regions represent the sites predicted by ProSiteHunter.

**Table S16** Analyze the impact of fine-tuning with sub-MSAs of varying depths on the performance of the protein-protein binding site prediction task (Test70).

| **MSA** | **Sequence Number** | **ROCAUC** | **PRAUC** | **F1** | **MCC** |
| --- | --- | --- | --- | --- | --- |
| No fine-tuning | 0 | 0.757 | 0.446 | 0.467 | 0.321 |
| Top1 | 351 | 0.775 | 0.471 | 0.489 | 0.339 |
| Top25 | 7436 | 0.780 | 0.474 | 0.491 | 0.342 |
| Top50 | 14690 | 0.783 | 0.483 | 0.499 | 0.355 |
| Top75 | 21850 | 0.781 | 0.475 | 0.493 | 0.350 |
| Top100 | 28934 | 0.778 | 0.473 | 0.487 | 0.346 |
| Top200 | 56740 | 0.774 | 0.474 | 0.486 | 0.344 |

As shown in **Fig. S10**. Focal loss mitigates class imbalance by down-weighting easy samples and emphasizing harder ones (with an approximate weighting ratio of 1:3 in our implementation), thereby encouraging the model to focus more on minority-class residues (i.e., binding sites), which is well aligned with the highly imbalanced nature of this task.

Experimental results demonstrate that Focal loss consistently improves performance across different tasks, with more pronounced gains in metrics that are sensitive to class imbalance. For example, in the Protein-DNA task, PRAUC increases from 0.485 to 0.511 (approximately 5.4%), and MCC improves from 0.473 to 0.486 (approximately 2.7%). Similarly, in the Protein-RNA and Protein-Protein tasks, although ROCAUC shows only marginal changes, PRAUC, F1-score, and MCC exhibit more substantial improvements, indicating enhanced recognition of minority-class residues.


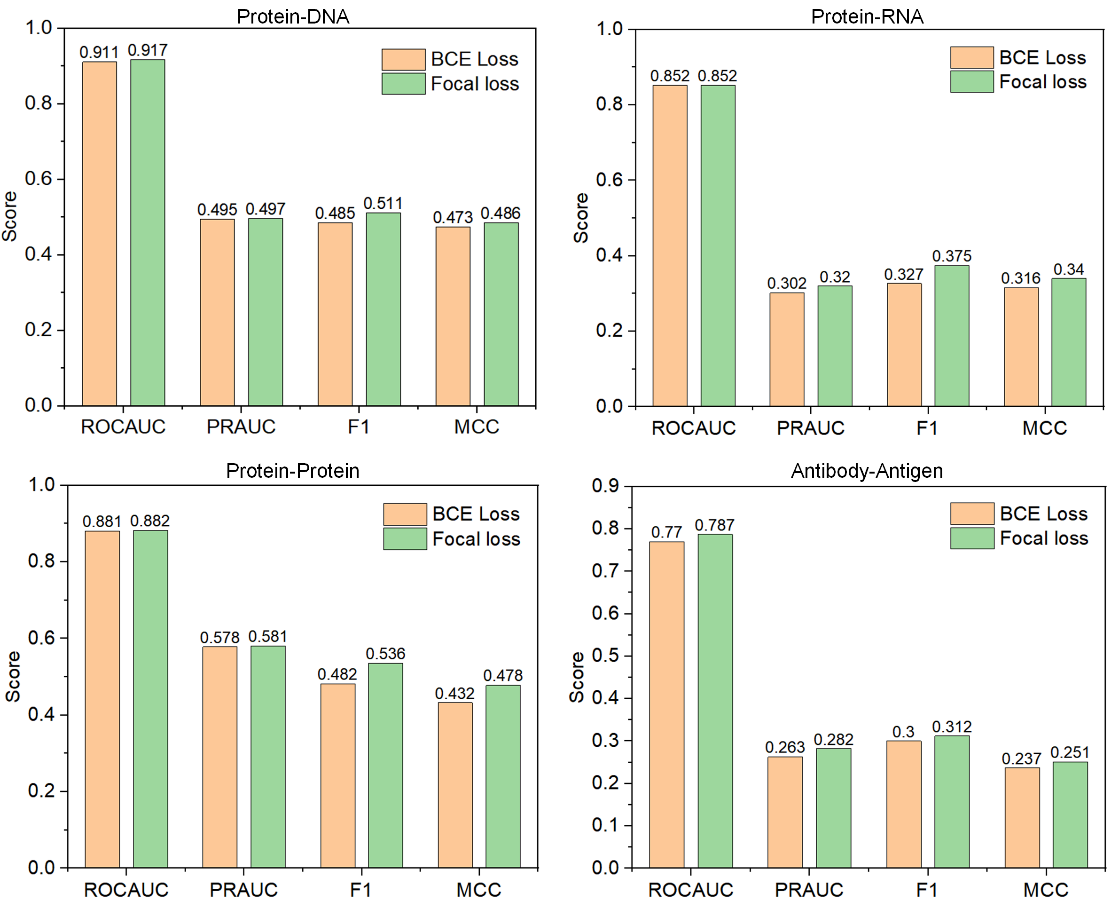


**Fig. S10** Performance comparison of ProSiteHunter using Focal loss and binary cross-entropy loss across four site prediction tasks.

### **Evaluation metrics**

The protein-macromolecule binding sites prediction task is a binary classification task, therefore, we followed previous studies and used recall, precision, F1 score, and Matthew correlation coefficient (MCC) as metrics to evaluate the performance of our method. The calculation formula is as follows:

$\mathrm{Recall}$: The proportion of positive examples correctly identified by the model to all positive examples.

$$\begin{aligned} Recall=\frac{TP}{TP+FN}\#(1) \end{aligned}$$

$\mathrm{Precision}$: The proportion of positive cases predicted by the model to the true positive examples.

$$\begin{aligned} Precision=\frac{TP}{TP+FP}\#\left( 2 \right) \end{aligned}$$

$F1$: The harmonized average of precision and recall.

$$\begin{aligned} F_{1}=2\times\frac{Precision\times Recall}{Precision+Recall}\#\left( 3 \right) \end{aligned}$$

$\mathrm{MCC}$: The correlation coefficient of all prediction results is combined, and the value range is [-1, 1], where 1 indicates a perfect prediction.

$$\begin{aligned} MCC=\frac{TP\times TN-FP\times FN}{\sqrt{\left( TP+FP \right)\left( TP+FN \right)\left( TN+FP \right)\left( TN+FN \right)}}\#\left( 4 \right) \end{aligned}$$

where $TP,FN,TN,FP$ represent the number of true positives, false negatives, true negatives, and false positives, respectively. In particular, when the ratio of positive and negative samples is not well balanced, F1 and MCC are more objective indicators of model performance than recall or precision alone, as the binding sites are usually much smaller than the non-binding sites (positive and negative sample imbalance). In addition, we also used ROCAUC and PRAUC to evaluate the model from the perspective of global threshold changes, where PRAUC is more sensitive to class imbalance and is a key indicator of binding-site prediction.
